# Improved pulmonary embolism detection in CT pulmonary angiogram scans with hybrid vision transformers and deep learning techniques

**DOI:** 10.1038/s41598-025-16238-4

**Published:** 2025-08-26

**Authors:** Abeer Abdelhamid, Amir El-Ghamry, Ehab H. Abdelhay, Mohammed M. Abo-Zahhad, Hossam El-Din Moustafa

**Affiliations:** 1 Electronics and Communications Engineering Department, Faculty of Engineering, Mansoura University, Mansoura, 35516 Egypt; 2 Computer Science Department, Faculty of Computers and Information, Mansoura University, Mansoura, 35516 Egypt; 3 Electrical Engineering Department, Faculty of Engineering, Sohag University, Sohag, 82524 Egypt

**Keywords:** Computed Tomography (CT), Deep Learning (DL), Discrete Wavelet Transforms (DWT), Pulmonary Embolism (PE), Vision Transformers (ViT), Image processing, Machine learning

## Abstract

Pulmonary embolism (PE) represents a severe, life-threatening cardiovascular condition and is notably the third leading cause of cardiovascular mortality, after myocardial infarction and stroke. This pathology occurs when blood clots obstruct the pulmonary arteries, impeding blood flow and oxygen exchange in the lungs. Prompt and accurate detection of PE is critical for appropriate clinical decision-making and patient survival. The complexity involved in interpreting medical images can often results misdiagnosis. However, recent advances in Deep Learning (DL) have substantially improved the capabilities of Computer-Aided Diagnosis (CAD) systems. Despite these advancements, existing single-model DL methods are limited when handling complex, diverse, and imbalanced medical imaging datasets. Addressing this gap, our research proposes an ensemble framework for classifying PE, capitalizing on the unique capabilities of ResNet50, DenseNet121, and Swin Transformer models. This ensemble method harnesses the complementary strengths of convolutional neural networks (CNNs) and vision transformers (ViTs), leading to improved prediction accuracy and model robustness. The proposed methodology includes a sophisticated preprocessing pipeline leveraging autoencoder (AE)-based dimensionality reduction, data augmentation to avoid overfitting, discrete wavelet transform (DWT) for multiscale feature extraction, and Sobel filtering for effective edge detection and noise reduction. The proposed model was rigorously evaluated using the public Radiological Society of North America (RSNA-STR) PE dataset, demonstrating remarkable performance metrics of 97.80% accuracy and a 0.99 for Area Under Receiver Operating Curve (AUROC). Comparative analysis demonstrated superior performance over state-of-the-art pre-trained models and recent ViT-based approaches, highlighting our method’s effectiveness in improving early PE detection and providing robust support for clinical decision-making.

## Introduction

Pulmonary embolism (PE) is a serious condition where blood clots block pulmonary circulation, obstructing the pulmonary arteries or their branches^1^. The most frequent underlying cause of PE is deep vein thrombosis (DVT), typically originating as a thrombus in the deep veins of the lower limbs^2^. PE is most commonly caused by a clot in another region of the body, usually a leg or an arm. The restricted blood flow increases the blood pressure, which decreases the oxygen levels in the blood^3,4^. When the right ventricle distributes oxygenated blood to the lungs, blood clots in pulmonary arteries obstruct its flow and can even migrate into the lungs. It is a clot of blood that forms in the pulmonary arteries of the pulmonary arteries. Clinically, PE is the third most common cardiovascular emergency, following heart attacks and strokes^5^. The clinical manifestations of PE range considerably, from asymptomatic or mild symptoms to severe presentations that may rapidly progress to shock or cardiac arrest. Typical symptoms include sudden-onset shortness of breath (dyspnea), pleuritic chest pain, tachycardia, and clinical indicators of right ventricular strain^6^. Severe complications, such as ischemia and right-sided heart failure, further exacerbate patient prognosis^7^. The variability and non-specific nature of PE symptoms significantly complicate accurate clinical diagnosis, often causing delays in appropriate management and treatment decisions. These challenges primarily arise from the heterogeneity of patient presentations, the variability of hemodynamic responses, and underlying conditions that can mask or mimic PE symptoms^8^.

The global epidemiological burden of PE is substantial, with approximately 115 cases per one hundred thousand people diagnosed annually in the United States. Untreated severe cases have a notably high mortality rate, estimated between 18% to 65%^9^. Moreover, around 34% of PE-related deaths occur suddenly, before diagnosis or commencement of appropriate treatment, emphasizing the critical need for early detection and intervention^10^. PE is typically classified based on anatomical location–such as right-sided, left-sided, or central–and according to the temporal development–acute PE forming rapidly, or chronic PE forming progressively over time^11^. Currently, Computed Tomography Pulmonary Angiography (CTPA) serves as the primary diagnostic tool for PE, offering reliable results above 90% for the accurate detection and localization of emboli^12^. The procedure involves intravenous administration of iodinated contrast medium, enabling radiologists to visualize emboli as dark filling defects within the pulmonary arteries^13^. Despite its advantages, manual interpretation of CTPA remains challenging due to the sheer number of images (typically between 300-500 slices per scan) that must be thoroughly examined for subtle abnormalities^14^. This requirement not only imposes considerable time and workload constraints on radiologists but also significantly increases the risk of human error and subsequent diagnostic delays^15,16^. Moreover, the accuracy of traditional diagnostic methods is further constrained by ambiguous clinical symptoms, limited specificity of biomarkers (e.g., D-dimer), and inadequate availability of specialized imaging across healthcare facilities^17^.

Advancements in artificial intelligence (AI) and deep learning (DL) techniques offer promising opportunities to address these diagnostic limitations by developing automated, efficient, and accurate Computer-Aided Diagnostic (CAD) systems. Over recent decades, various approaches have been explored, including deterministic image processing and probabilistic/statistical models leveraging machine learning (ML) algorithms^18^. Among these, convolutional neural networks (CNNs) have emerged as particularly effective for medical image analysis, successfully automating disease detection tasks with considerable accuracy^19^. Recently, vision transformers (ViTs), initially designed for Natural Language Processing (NLP), have rapidly gained recognition in medical imaging due to their superior performance in capturing long-range dependencies within images, substantially enhancing classification and segmentation accuracy^20^. Recent advances in medical image analysis have leveraged hybrid ViT models combined with recurrent networks to improve medical image analysis. For example, Ahmed et al.^21^ proposed a hybrid ViT-GRU model for brain tumor detection and classification in MRI scans, incorporating explainable AI techniques to enhance interpretability. Similarly, Hossain et al.^22^ suggested a ViT-LSTM model for brain stroke detection and classification in CT images, also utilizing explainable AI techniques to support clinical decision-making. Despite these substantial advancements, DL-based methods for automated PE detection still face significant challenges, particularly concerning high false-negative rates and limited capability in simultaneously capturing local and global image contexts. Additionally, practical diagnostic procedures for PE extend beyond mere detection to include accurately classifying embolism location, determining its nature (acute versus chronic), and calculating clinically relevant metrics such as the right-to-left ventricular (RV/LV) ratio^23^. Developing a robust and comprehensive automated diagnostic method remains complicated due to variations in patient anatomy, artifacts related to patient movement and breathing, inconsistent contrast medium distribution, and comorbidities that obscure diagnostic clarity^24^.

To overcome these challenges, our study aims to develop an AI-based framework specifically designed for accurate, efficient, and early detection of PE from CTPA images. We introduce a sophisticated hybrid preprocessing pipeline incorporating an autoencoder (AE) for dimensionality reduction, data augmentation to avoid overfitting, discrete wavelet transform (DWT) for multilevel feature extraction, and Sobel edge detection for enhanced image clarity. The classification stage employs an ensemble learning strategy combining the complementary strengths of three deep neural networks: ResNet50, DenseNet121, and Swin Transformer, effectively capturing both local details and global context. The primary contributions of this research include:

- Development an advanced ensemble classifier combining ResNet50, DenseNet121, and Swin Transformer architectures, effectively capturing both local details and global contextual information to significantly enhance diagnostic accuracy and reliability.
- Proposed an innovative hybrid preprocessing pipeline that integrates AE-based dimensionality reduction, data augmentation to avoid overfitting, DWT for multiscale feature extraction, and Sobel filtering for edge enhancement, in order to improve robustness in PE detection.
- Rigorously validated the proposed methodology on RSNA-STR dataset, achieving superior performance with an accuracy of 97.80% and 0.99 for AUROC.
- Demonstrated substantial performance improvement over state-of-the-art (DL) and ViT-based approaches through extensive comparative analysis, underscoring the clinical applicability and potential of the proposed model for automated PE diagnosis.

The structure of the rest of this paper is organized as follows: Sect. “Related Work” provides an overview of relevant studies, highlighting recent advancements and existing limitations in automated PE detection using DL and ViT methods. Section “Materials and Methods” presents the detailed methodology of our proposed approach, outlining the hybrid preprocessing techniques, the ensemble classification framework, and the specifics of our experimental setup. Section “Experimental Results” reports the experimental results, including comprehensive performance evaluation metrics and comparative analyses with state-of-the-art methods. Section “Discussion” provides a discussion on the implications, strengths, limitations, and practical considerations of our proposed method. Finally, Sect. “Conclusions and Future Directions” concludes the paper and suggests potential directions for future research.

## Related work

PE is a challenging disease to diagnose clinically because it overlaps symptoms with a variety of different ailments. The continuous improvements of DL and enhanced computer hardware has greatly advanced computer-based medical imaging diagnosis. However, there are studies that can assist in addressing the issue, some of which are included below. Particularly for disease diagnosis, ML is a robust algorithm generally considered to be effective for medical purposes. It is more cost-effective than standard medical checks. Tajbakhsh et al.^25^ utilized a CNN for the detection of PE in CTPA images. They applied their approach on 121 CTPA images that included data from 326 patients. Their suggested method demonstrated superior performance compared to standard ML algorithms in recognizing individual emboli, with a sensitivity of 83%. Ma et al.^26^ suggested a two-phase multitask learning algorithm that for detecting PE and its parameters, including the location (acute or chronic) and the RV/LV ratio, reducing false negatives. Using their method on RSNA dataset, their model obtained promising performance on the hold-out test set, with a window-level AUROC of 0.93, a sensitivity of 0.86. Khan et al.^27^ created a DL model utilizing CNN for PE identification and classification on RSNA dataset. They employed an Xception model for extracting features, which were then used for classification through transfer learning. Their technique achieved an accuracy rate of more than 90%.

Moreover, Islam et al.^28^ conducted an analysis on DL for diagnosing PE using RSNA dataset. They contrasted self-supervised learning with supervised learning and evaluated the performance of CNNs against ViTs at the image level. For performance evaluation, the authors employed several CNN models and reported AUC scores of 0.86 and 0.89 for Resnet50 and SeXception. Yuan et al.^29^ presented a PE detection approach based on the upgraded faster region-based CNN (Faster R-CNN), entitled more Accurate Faster R-CNN (MA Faster R-CNN). A new feature fusion network, the Multi-scale Fusion Feature Pyramid Network (MF-FPN), was proposed by extending and adding two bottom-up channels to the Feature Pyramid Network (FPN) for feature extraction enhancement. They applied their method on a dataset contained 7771 CTPA images, and reached an average accuracy of 85.88%. Huhtanen et al.^30^ created a CNN architecture based on InceptionResNet V2 with Long-Short-Term-Memory (LSTM) network to analyze CTPA stacks as slice sequences. They used dataset consisted of 800 CTPA samples. Their method achieved specificity 90.7% and sensitivity 83.5%) respectively. Huang et al.^31^ created PENet, a DL model for end-to-end solution PE detection in volumetric CTPA scans. It is a 77-layer 3D CNN initially trained on Kinetics-600 dataset and fine-tuned with a retrospective CTPA dataset gathered from one institution. Their method scored sensitivity and specificity of 73% and 82%, respectively. PE-DeepNet is a hybrid DL-CNN model that can identify PE quickly and accurately was developed by Lynch et al.^32^. They used RSNA dataset for their experiment and achieved an accuracy of 94.2%.

In contrast, Djahnine et al.^33^ developed a DL-based strategy to detect and quantify the severity of PE by integrating the Qanadli scoring with right-to-left ventricle diameter ratio. For training and validation, they utilized multiple datasets including a 3D CTPA collection from 1268 individuals with image-level annotations, as well as two additional datasets: CAD-PE (91 patients) and FUME-PE (35 patients). Performance metrics indicated robust classification capabilities, with the model attaining an AUC of 0.870 (95% confidence interval [CI]: 0.850-0.900) on training data and 0.852 (95% CI: 0.810-0.890) on test data. Guo et al.^34^ employed feed-forward and elman back propagation techniques on 294 cases using a modified neural network and a perfusion scan for diagnosis. Their strategies achieved an accuracy of 93.23% and 86.61%, respectively. Wu et al.^35^ proposed a model Self attention mechanism prioritizes YOLO (PE-YOLO) on RSNA dataset for PE identification. Their strategy reported an accuracy of 86.4% and sensitivity of 90.7%. Suman et al.^36^ suggested a hybrid model that combines a two-stage attention with CNN and LSTM to predict PE in CT scans. Their proposed approach outperformed both the CNN model and the single stage CNN-LSTM network, scoring an AUC of 0.95. Condrea et al.^15^ utilized a dual-hup neural network on RSNA dataset. Their method achieved sensitivity and specificity of 92%, 96.1% respectively. ViT-based multi-task learning model combining EfficientNet-B7 and a self attention enhanced ViT was proposed by Mohammed et al.^20^ for PE detection, localization, and classification. It achieved an AUC of 96.83% for positive PE on RSNA dataset. A multimodal DL model combining CTPA scans with clinical data was proposed by Cahan et al.^37^ for automatic PE risk stratification. Using CNN and TabNet transformer with bilinear attention, their method achieved 0.96 AUC, 90% sensitivity, demonstrating the impact of multimodal fusion with minimal annotation. Recently, most studies used CNN models while recent studies explore ViTs ane ensemble combining both for better performance. Advanced preprocessing techniques have also helped improve results. However, challenges remain including generalizing these models to different datasets, handling imbalanced datasets, representing complex medical image features, ensuring models are interpretable by clinicians, and maintaining computational efficiency. Additionally, integrating clinical data with medical images is an important trend to enhance diagnosis. Based on these points, the current research aims to improve diagnostic accuracy while addressing these challenges. In conclusion, both ViTs and CNN-based approaches demonstrate considerable potential in PE detection in CTPA scans, with ongoing efforts focused on further improving their performance and interpretation. Although the previous studies have applied different DL techniques to enhance diagnostic accuracy in CT scans, many still face challenges in generalizing the models. In addition, some models fail to adequately address computational efficiency.

## Materials and methods

The integration of imaging technologies into clinical workflows has long supported accurate diagnosis and effective treatment planning by healthcare professionals. The Radiological Society of North America (RSNA)^38^ has joined forces with the Society of Thoracic Radiology (STR)^39^. Five international research centers donated CTPA images, and over 80 qualified thoracic radiologists labeled them with through clinical annotations. It comprising more than 12,000 CT studies^39^. At both the image and exam levels, the dataset provides annotations that identify whether or not PE is present in each scan. The images are stored in DICOM format, a system commonly used for digital medical imaging. Modern CT scanners employ ’Spiral CT’, where the collected data is reconstructed to generate a 3D volume. These volumes can be sliced digitally to view different planes, with CT numbers used to adjust gray levels and enhance image structures^40,41^. As the dataset contains slices from the middle of the body which offers the best alignment of the lungs with the CTPA sensor, only a small percentage (5%) of the scans that identified PE actually contained verified instances of the condition.

### Data preprocessing

**Fig. 1:**
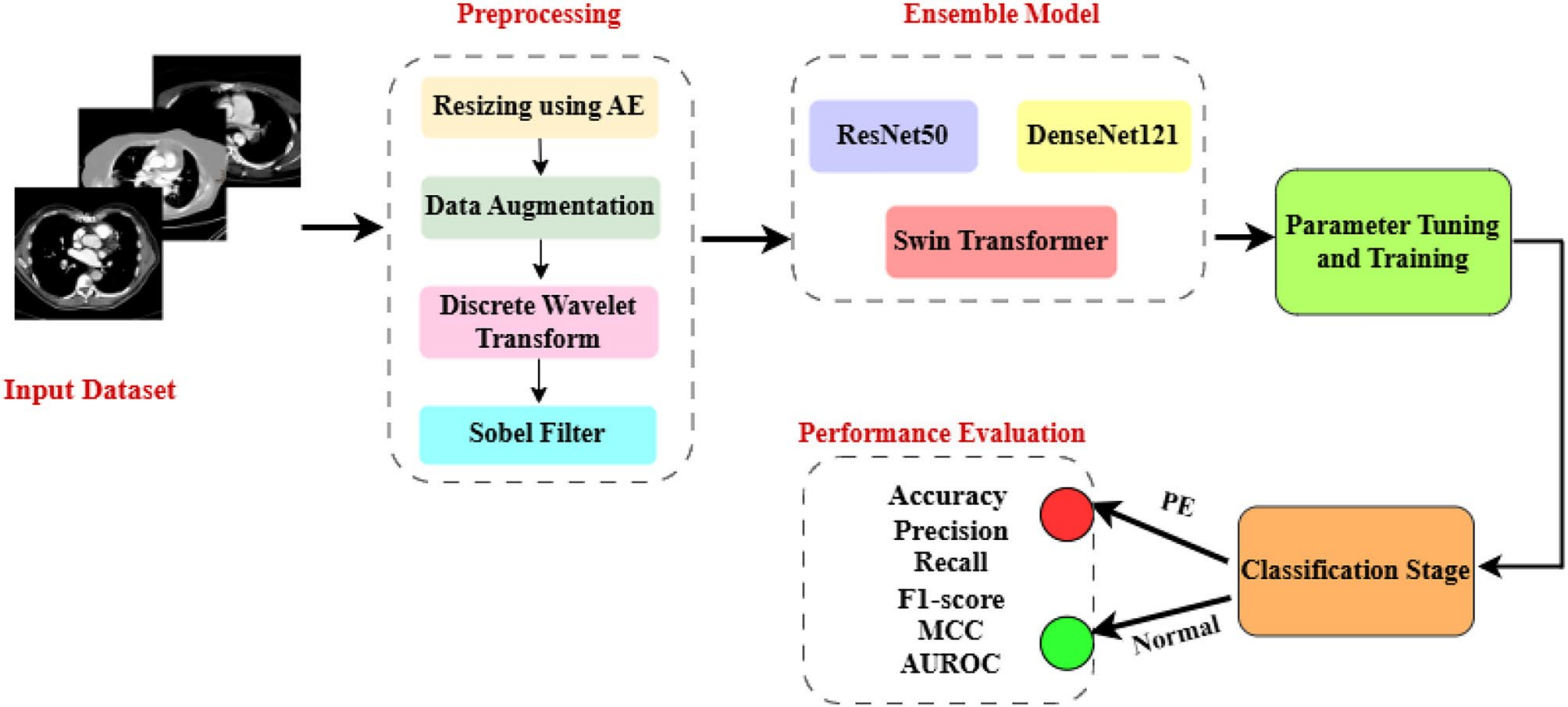
Blockdiagram of the proposed pipeline for PE diagnosis.

**Fig. 2:**
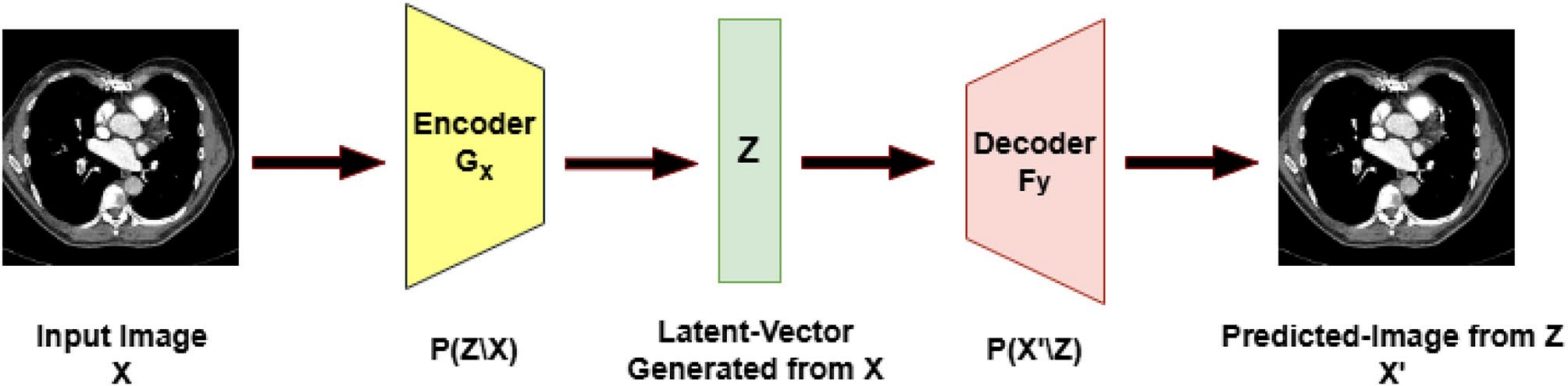
Blockdiagram of Auto Encoder Module.

We utilize the public available RSNA-STR dataset in the proposed model. This dataset includes annotations for different types of PE (central, segmental, subsegmental). For this study, thee classification task was formulated as a binary decision detecting only the precense or absence of PE. The provided dataset contains $512\times 512$ DICOM CTPA images imaged at the arterial phase. We provide these scans to our module after converting the DICOM images to grayscale images. The used dataset was split into 70%:15%:15% for training, test, and validation sets using a patient-level split, such that no patient appears in more than one subset thereby preventing the leakage. The system sequentially conducts input scaling, data preprocessing, model creation, and classification. The first step is encoding, a method used in ML to convert categorical variables into numerical/digital values that can be conveniently accessed^42^. Autoencoders (AE), a multi-layered feed-forward neural network, is an unsupervised machine learning strategy for reducing dimensionality in multivariate data sets^43^. Figure 1 explains the structure of the proposed model. In the proposed model, we use auto encoder to easily encode the image dataset, followed by auto decoder to reconstruct the processed image that was compressed throughout the encoding process. An AE is a simple feed-forward network where information is fed to the input layer and then transferred through hidden levels. Each has a different amount of nodes/neurons that transform the input and produce the output. The nodes are expanded into multiple layers, each of which is connected to all of the nodes from the previous layers. Our system includes a learnable input scaling module that resizes images to a fixed size of $224\times 224\times 3$ using an auto-encoder design. The AE is composed of a symmetrical structure consisting of three convolutional layers in the encoder, followed by a bottleneck layer representing the latent feature space (with 128 dimensions), and the three deconvolutional layers in the decoder. This structure enables effective compression of the input images while preserving spatial features necessary for accurate classification. We have also clarified that the AE not only serves for image resizing to (224 x 224 x 3), but also performs dimensionality reduction by extracting key semantic features, thus reducing computational load for subsequent classifiers in the ensemble. This balance helps avoid overfitting and improves model robustness. Figure 2 shows a general illustration of AE. In response to class imbalance within the dataset, data augmentation methods are employed as a subsequent step. The objective of data augmentation is to enhance the training set by generating additional images that retain comparable patterns, thereby improving the model’s performance. A summary of the data augmentation techniques applied can be found in Table1.

**Table 1:** Data augmentation processes which were applied on the used dataset.

**Process**	**Value**
Rotation range	20
Width shift range	0.2
Height shift range	0.2
Shear range	0.2
Zoom range	0.2
Vertical flip	True
Horizontal flip	True
Gaussian blur	$\sigma =2$
Fill mode	Nearest

#### Discrete Wavelet Transform (DWT)

Wavelet transforms evaluate images in many resolutions and identify minor oscillations at multiple levels, making them effective tools for enhancing contrast. Effective contrast augmentation should consider both global and local information^44^. As an effective technique for image processing, the discrete Wavelet transform (DWT) operates in both the time and frequency domains and offering enhanced capabilities for analyzing localization indicators^45^. After AE and data augmentation stages, we employed DWT. The image is split into four subbands, each containing different coefficients: *LL, HH, HL, and LH* Where:

***LL(approximation subband):*** includes low frequencies and gives a simplified version of the image, keeping its main features.

***LH (Horizontal detail sub-band):*** captures low frequencies horizontally and high frequencies vertically, emphasizing vertical edges in the image.

***HL (Vertical detail sub-band):*** highlights horizontal edges by using high frequencies horizontally and low frequencies vertically.

***HH (Diagonal detail sub-band):*** uses high frequencies in both directions, enhancing diagonal edges and small details.

These coefficients may contain noise or essential signal features. Smaller coefficients are typically associated with noise, while larger ones correspond to key signal characteristics. The wavelet thresholding method utilizes a threshold function to filter out smaller coefficients in the *HH, HL, and LH* subbands^46^. Thresholding techniques can be classified into two types: soft thresholding and hard thresholding. In soft thresholding, wavelet coefficients that are less than or equal to threshold T are set to zero, while those greater than T are reduced towards zero by the value of T. In hard thresholding wavelet coefficients above the threshold T are preserved, sitting it apart from the soft thresholding method. Two common shrinkage approaches for thresholding are VisuShrink which estimates one threshold for the image and BayesShrink which calculates a threshold for each subband^47^. The mathematical expression for the two-dimensional DWT of an image $I(x,y)$ is given by^48^:

1 $$\begin{aligned} I_x(x, y) = \sum _{a=-\infty }^{\infty }\sum _{b=-\infty }^{\infty }I(x, y)g(x-2a)g(y-2b) \end{aligned}$$

2 $$\begin{aligned} I_y(x, y) = \sum _{a=-\infty }^{\infty }\sum _{b=-\infty }^{\infty }I(x, y)h(x-2a)g(y-2b) \end{aligned}$$

3 $$\begin{aligned} I_d(x, y) = \sum _{a=-\infty }^{\infty }\sum _{b=-\infty }^{\infty }I(x, y)g(x-2a)h(y-2b) \end{aligned}$$

4 $$\begin{aligned} I_h(x, y) = \sum _{a=-\infty }^{\infty }\sum _{b=-\infty }^{\infty }I(x, y)h(x-2a)h(y-2b) \end{aligned}$$

here $I_x(x, y)$, $I_y(x, y)$, $I_d(x, y)$, and $I_h(x, y)$ are the approximation and detail coefficients at different resolutions. In addition, g(n) and h(n) refer to the wavelet filters, while x and y represent the image coordinates. The goal of DWT technique is to improve the image’s fine details by the high frequency components. This improve the image’s clarity and sharpness, as well as the small details such as edges and components.

#### Edge detection

**Fig. 3:**
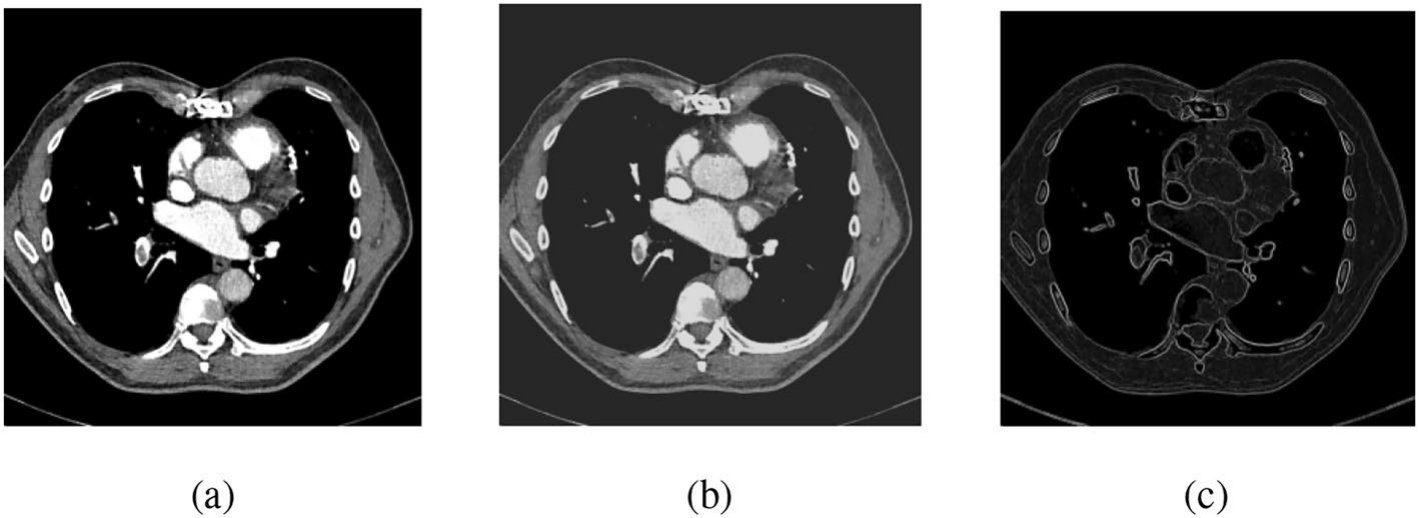
Effect of applying preprocessing techniques on the dataset where (**a**) Original image, (**b**) Image after DWT, and (**c**) Image after Sobel filter.

Edge detection methods often employ Sobel operator. Serving as a discrete differentiation tool, this operator approximates the gradient of the image intensity function^49^. It is computationally efficient, using a small separable filter for convolution in both directions. The effect of the preprocessing techniques is observed in Figure 3. Sobel operator enhances image clarity by extracting and sharpening edges and contours^50^. However, it creates a rough gradient approximation, especially for images with large frequency changes^51^. Sobel operator includes two separate steps:

1- A triangle filter is used for smoothing, applied perpendicular to the derivative direction.

2- Two convolution kernels are used in the derivative direction to apply the central difference, detecting edges a gradient- based method^52^. The gray value at each point is derived by combining the horizontal and vertical intensity components, typically using the euclidean norm. The gradient direction is then estimated using the arctangent of the vertical-to-horizontal intensity ratio.

### Classification stage

For the classification task, an ensemble model was employed, combining three deep neural networks (Resnet50, Densenet121, and Swin Transformer). The primary goal is to enhance performance in the task of PE classification from CT images. These networks were selected based on their established effectiveness in extracting complex features from the dataset, making them particularly suited for the classification task.

***1. Resnet50:*** known for its deep architecture and the ability to improve feature extraction through residual learning, which helps in overcoming the vanishing gradient problem.

***2. Densenet121:*** It employs a dense connectivity structure, where each layer is directly linked to all preceding layers, promoting more efficient learning and feature reuse. Thus boosting the model’s accuracy.

***3. Swin transformer:*** a transformer model designed to handle high- dimensional data and capture long range dependencies and context within the image.

The advent of transformers has revolutionized computer vision (CV) over the past decade. Initially developed for natural language processing tasks, transformers have proven highly effective in dealing sequential data. ViT model improves upon CNN by using fixed-size patch embeddings processed through a transformer encoder with a self-attention mechanism^53^. In contrast to CNNs’s fixed receptive fields, ViTs’s self-attention evaluates each patch’s importance dynamically. This leads to enhancing the ability to understand complex visual relationships and identify detailed features in images. The main elements of the ViT model are:

***1. Patch Embedding:*** The image is separated into (16$\times$16) pixels, and converted into vectors.

***2. Positional Encoding:*** Spatial information is added to the patches to help the model understand their order.

***3. Transformer Encoder:*** Composing multi-head self-attention (MSA) layers and feed-forward networks, the model captures long-range dependencies and global context.

***4. Classification Head:*** Once tokens pass through the encoder, they are arranged and the final output is predicted via a fully connected layer.

ViTs are more effective CNNs at capturing long-range relationships in images, making them a powerful tool for image processing. Compared to CNNs, that use convolutions to focus on local characteristics, ViTs analyze the entire image simultaneously, allowing them to understand global context. The Swin Transformer has expanded its use beyond language processing, leading to advancements in CV^54^. Attention networks also improved models’ ability to prioritize important information in images. This attention mechanism is crucial for tasks needing accurate feature localization, which corresponded to the complexities of medical imaging, where small anomalies could easily be overlooked.

#### Shifted Window Transformer (Swin Transformer)

**Fig. 4:**
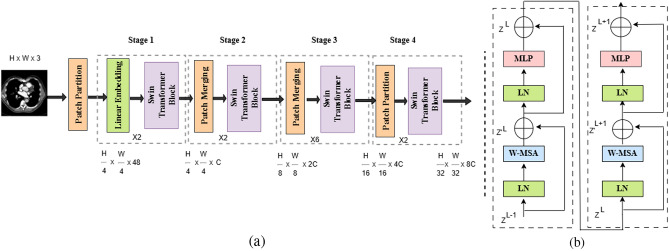
(**a**) Structure of Swin Transformer; (**b**) Two Successive Swin Transformer Blocks.

Swin Transformer is another excellent CV tool^55^. Figure 4 represents the structure of Swin Transformer. The patch partition layer begins by dividing the input image into smaller patches. The patch passes through a linear embedding layer, and a Swin Transformer block divided into four stages. Each stage repeats the linear embedding and Swin Transformer block which uses MSA, layer normalization (LN), and a two-later preceptron (MLP). By limiting self-attention to non-overlapping local windows and allowing cross-window interactions, the window-shift technique improves the model’s classification accuracy. In this study, the ensemble approach leverages the individual advantages of each structure, aiming to achieve more accurate and robust classification results. This ensemble approach based on stacking method, improves the performance by integrating predictions from multiple models and refining them through a higher level meta model. This can be represented mathematically as follows^56^:

5 $$\begin{aligned} E_{\text {meta}} = y\left( f_1(x), f_2(x), \dots , f_n(x)\right) \end{aligned}$$

where: $f_1, f_2, \dots , f_n$ represent the predictions of the base models for the input x, y is the meta model that integrates these predictions to produce the final output $E_{\text {meta}}$. In our ensemble approach, we employed stacking, wherein the output probabilities from the three base models (ResNet50, DenseNet121, and Swin Transformer) are concatenated and used as input features for meta learner, which is a logistic regression classifier.

## Evaluation metrics

To evaluate the performance of the proposed model, we employ accuracy, recall, specificity, and AUROC as the key metrics. To compute accuracy, the number of samples correctly classified is divided by the total number of samples. It can be calculated mathematically as shown in Eq 6 where, tp, tn, fp, and fn denote true positive, true negative, false positive and false negative, respectively^57^. In medical diagnosis, ignoring the positive category can lead to misdiagnosis and treatment delays. Therefore, having high score in both sensitivity or recall is crucial for effective medical image diagnosis. Precision is required for determining how many of the cases anticipated to be positive are actually positive. Precision is calculated by dividing the number of true positives by the sum of true positives and false positives as illustrated in Eq 7.

6 $$\begin{aligned} \text{ Acc }=\frac{tp+tn}{tn+tp+fn+fp} \end{aligned}$$

7 $$\begin{aligned} \text{ Pre }=\frac{tp}{tp+fp} \end{aligned}$$

Furthermore, the classifier uses sensitivity or recall to calculate the percentage of actual positive cases that are correctly identified as true positives. Eq 9 depicts the mathematical paradigm for the recall concept. The F1-score for all models is determined using their recall and precision as explained in Eq 10. Additionally, the Matthews Correlation Coefficient (MCC) is an effective statistic for assessing model classification difficulties. The value ranges from −1 to 1, with 1 being perfect prediction, 0 representing random prediction, and −1 representing full misprediction^58^. The MCC calculating formula is as follows:

8 $$\begin{aligned} MCC = \frac{tp \times tn - fp \times fn}{\sqrt{(tp + fp)(tp + fn)(tn + fp)(tn + fn)}} \end{aligned}$$

**Fig. 5:**
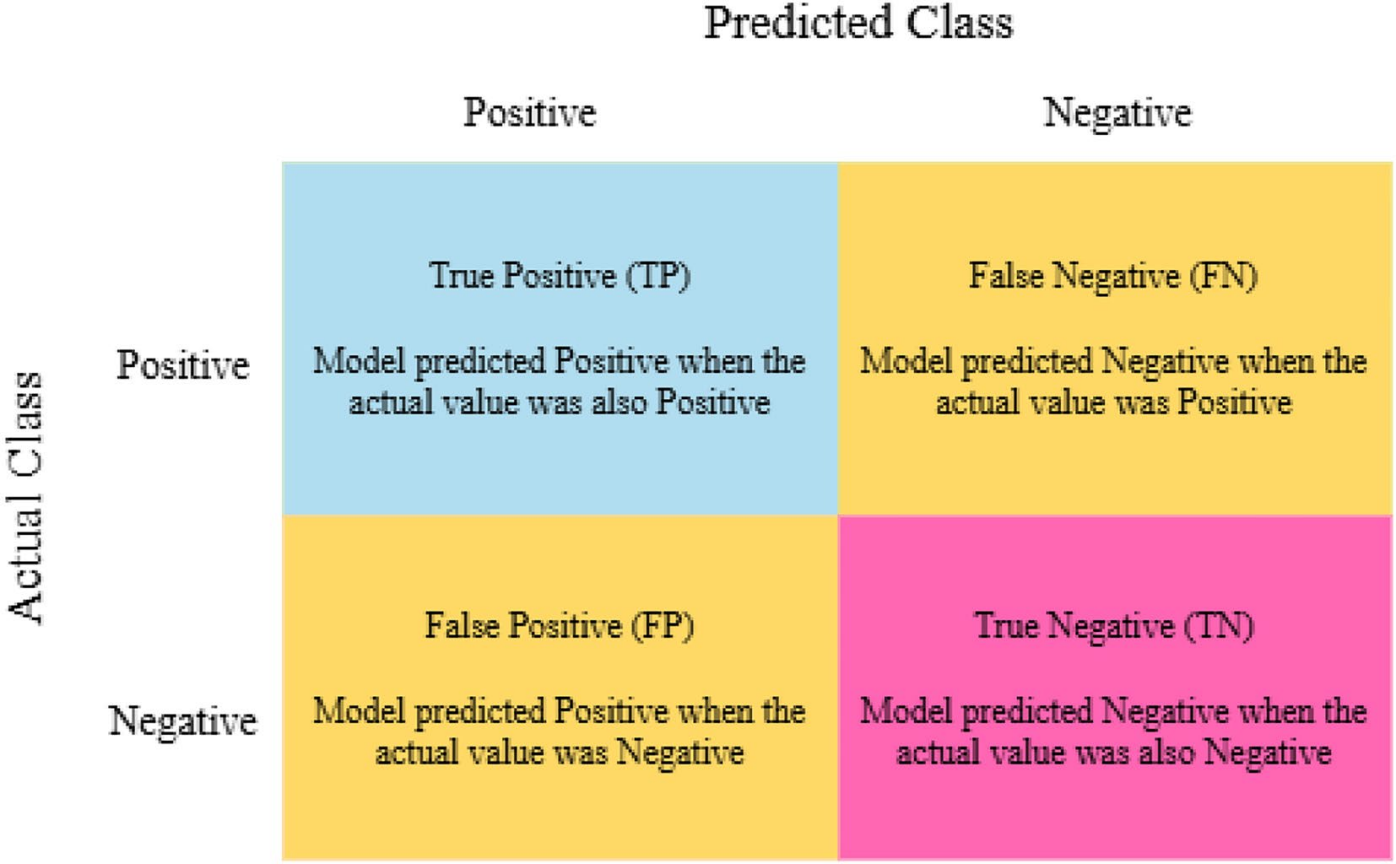
Confusion Matrix.

Additionally, confusion matrix helps evaluate how well a classification algorithm performs by obtaining both the correct and incorrect predictions it makes. It consists of two axis: true and predicted labels as shown in Fig. 5.

Also, the receiver operating characteristic (ROC) curve was calculated by comparing the rate of false positives with the rate of true negatives. It is utilized to comprehend both deterministic signals of categorization sorting and computational modeling problems. The AUC (area under the ROC curve) evaluates the ROC curve’s effectiveness in classifying inputs. As the AUC value increases, the model’s classification performance improves. The AUC is determined for each model and finally, all models are compared to determine which one outperforms the other.

9 $$\begin{aligned} \text{ Rec }=\frac{tp}{tp+fn} \end{aligned}$$

10 $$\begin{aligned} F1-score = \frac{2tp}{2tp+fp+fn} \end{aligned}$$

## Experimentation and performance

This section outlines the experimental results of the proposed model on the dataset and compares them with other established models. In general, deep network fitting training on large datasets produces better experimental outcomes. We developed an ensemble model based on the combination between three classifiers (ResNet50, DenseNet121, and Swin Transformer) as a classifier for PE classification task. The model relies on combining the predicted outcomes from each network in a complementary manner, leveraging the strength of each individual model to enhance the overall performance. These networks were selected because they are effective at extracting features from complex dataset like CT images. Swin Transformer utilizes MSA, which is responsible for capturing relationships within the input image. We set the number of attention heads to 8. The model consists of four Swin Transformer blocks each comprising MSA followed by a feed forward network. The attention layer’s output is passed through a two layer feed forward network, with ReLU activation function in the first layer. After the input has passed through Swin Transformer blocks, the output is flattened and passed to the classification layer. To prevent overfitting, a dropout layer with a value of 0.3 was added after the feed forward layers. The sigmoid activation function is chosen for the final classification.

**Fig. 6:**
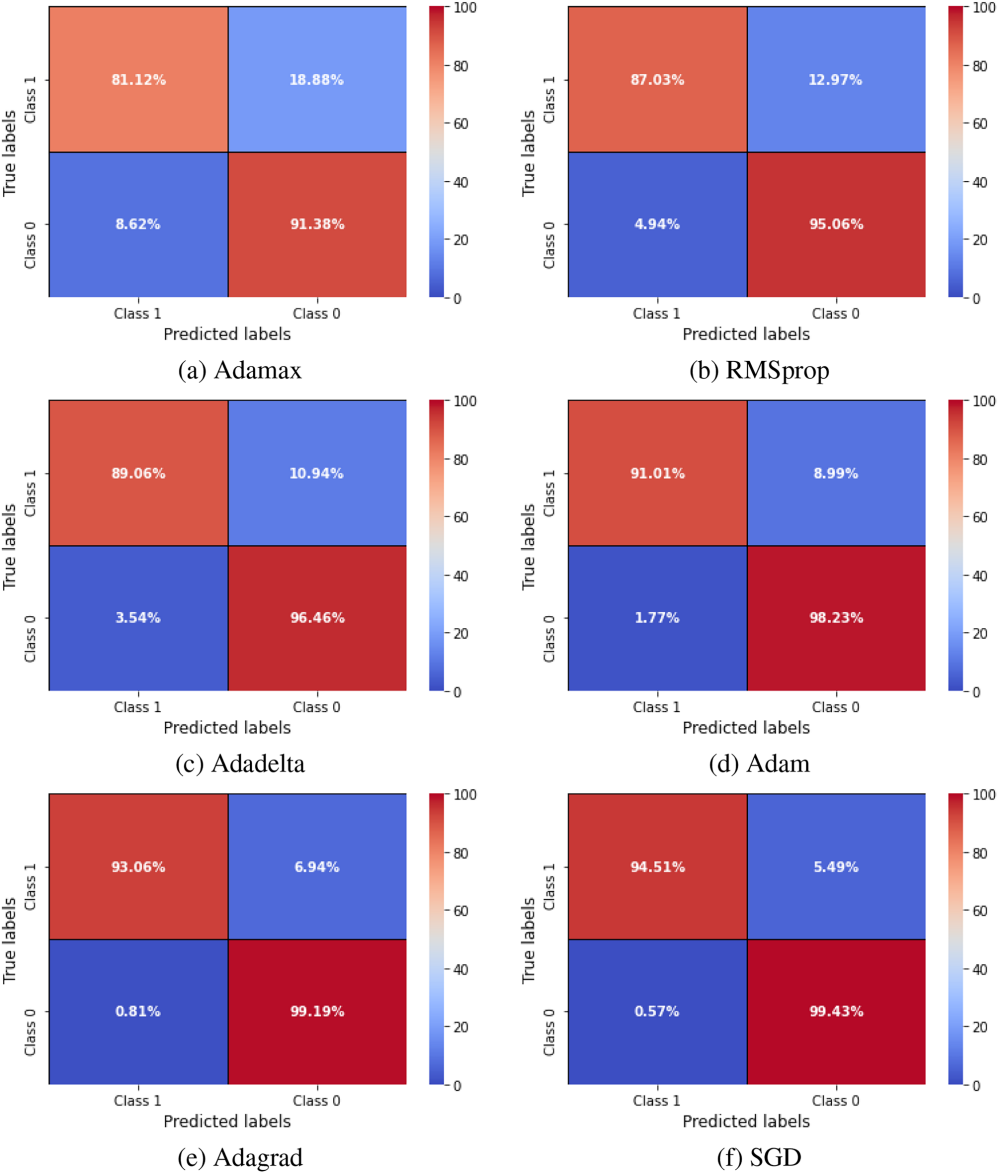
Confusion matrices depicting the performance of the proposed model using different optimizers.

**Fig. 7:**
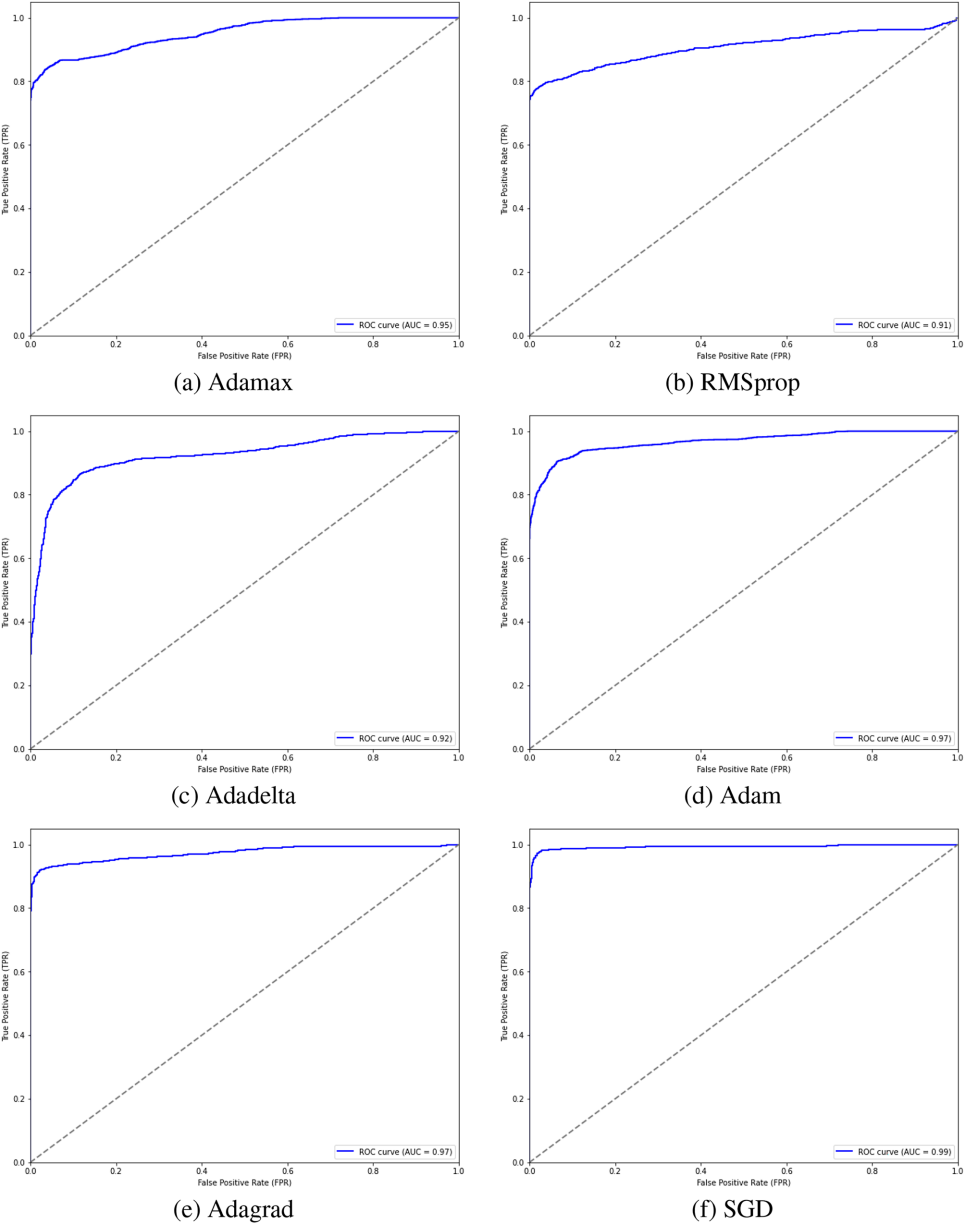
ROC curves illustrating the impact of different optimizers on the proposed model’s performance.

**Fig. 8:**
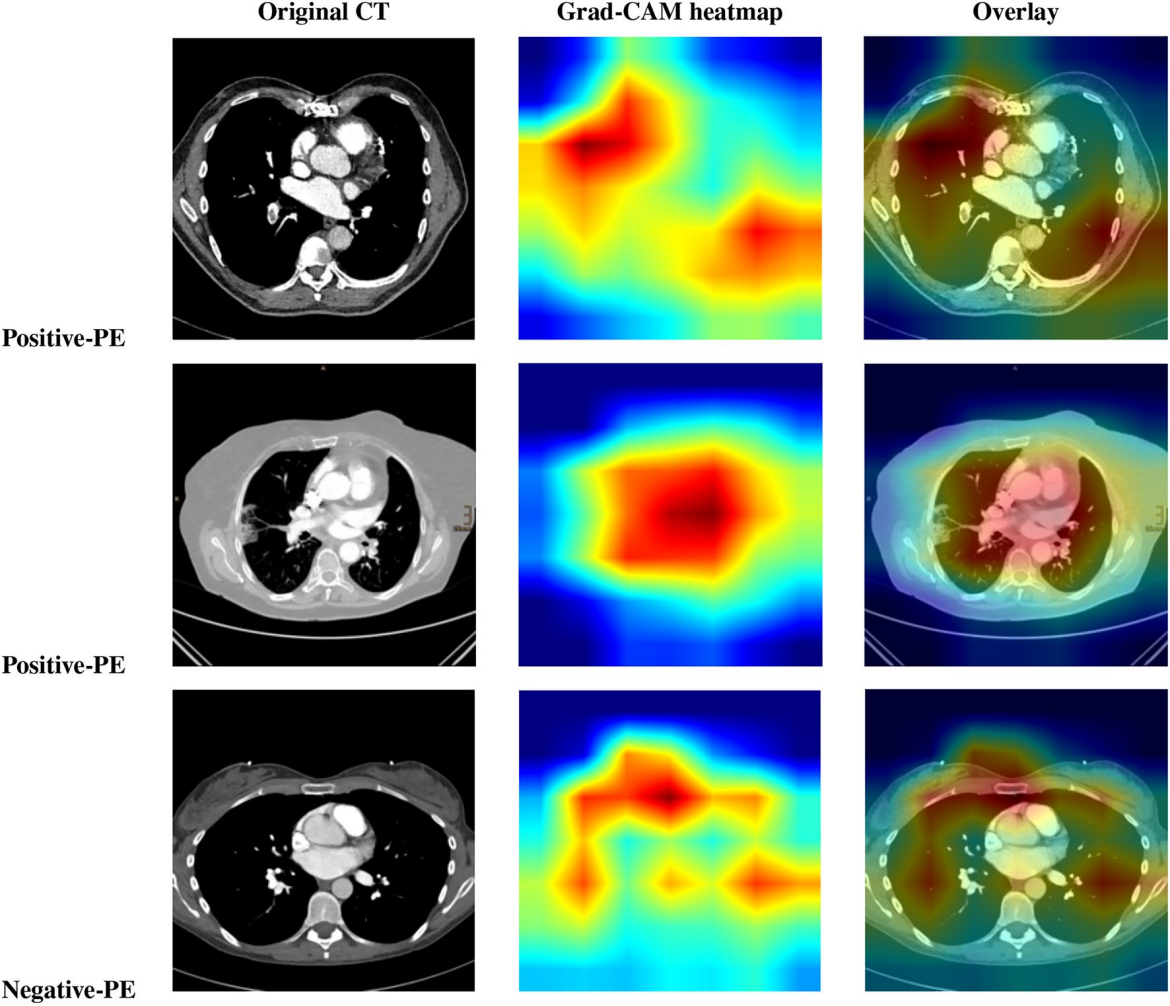
Grad-CAM visualizations of the proposed model on CT scans. Showing the original CT (left), the Grad-CAM heatmap (middle), and the overlay of heatmap on the original image (right). The first and second rows represent positive-PE cases, and the third row represents negative PE-case.

All experiments were conducted on NVIDIA T4 GPU with 16 GB and 12 GB RAM using Python 3.8 and Tensorflow 2.14.0 framework. The hyper-parameters were set to $10^{-5}$ for learning rate, batch size of 32, SGD optimizer, 20 epochs, and binary cross entropy loss function in the training step. Early stopping was applied based on validation loss to prevent overfitting and to ensure convergence. Several ablation studies were performed to assess the contribution of different system components to the overall performance. The first experiment involved comparing the proposed model with various CNN-based classifiers as shown in Table 2. Particularly, we tested different pre-trained models, including ResNet50, Vgg16, and DenseNet121 on the used dataset. The proposed model reached an accuracy of 97.80% surpassing the performance of the pre-trained models. In the second experiment, we applied different transformers like DeiT, PVT, and Swin Transformer as a classifiers on the used dataset. The performance of each model is shown in Table 3. In the third experiment, we evaluated the performance of the proposed model using different optimizers. Table 4 obtains the detailed results of the proposed model using different optimizers, while Figs. 6 and 7 display confusion matrices and ROC curves of the proposed model using different optimizers. As shown from Fig. 6, 94.51% of the PE samples were identified correctly, and 5.49% misclassified with SGD optimizer. It is clear that the outcome of the proposed model is influenced by the choice of optimizer. Furthermore, our technique yields promising results that are comparable to those of other DL studies for PE identification. Table 8 summarizes the results of the previous studies against the suggested technique. To demonstrate the interpretability of the proposed model, We used Grad-CAM visualizations to generate haetmaps from CT scans. As shown in Fig. 8 the highlighted regions correlate well with clinically relevant areas affected by PE, supporting the model’s focus on pathologically meaningful features. In addition, Table 5 obtains how different parts of the proposed model affect its performance by removing one part at a time. When Sobel filtering, DWT, or AE are removed, the model’s results get worse in all metrics. This means that each part helps improve the model. The full model with all parts works best, which shows that combining them gives the best results. Additionally, Table 6 displays the performance comparison between stacking and soft voting ensembles across 10 runs.To evaluate the statistical reliability of the model’s performance, we calculated the confidence intervals (CIs) for each metric using non-parametric bootstrapping with 1000 resamples drawn with replacement from the test set. For each metric, the 2.5th and 97.5th percentiles of the bootstrap distribution were used to define the lower and upper bounds of the 95% confidence interval. The resulting performance, along with the corresponding confidence intervals are summarized in Table 7. These intervals provide a robust indication of the model’s stability and performance variability across the dataset.

**Table 2:** Performance comparison of various CNN classifiers with the proposed model.

Model	ACC(%)	Pre(%)	Rec(%)	F1-score(%)	MCC
ResNet50	79.64	72.03	69.54	74.70	0.68
Vgg16	82.39	80.91	87.60	86.11	0.76
DenseNet121	85.00	81.27	79.34	83.45	0.82
**The proposed method**	**97.80**	**98.71**	**96.33**	**96.81**	**0.95**

**Table 3:** Results of different vision transformers as a classifiers against the proposed model. Here, Diet and PVT: stand for Data efficient Image Transformer and, Pyramid Vision Transformer respectively.

Model	ACC(%)	Pre(%)	Rec(%)	F1-score(%)	MCC
DeiT	88.64	86.15	84.56	83.94	0.79
PVT	92.34	90.92	89.06	88.57	0.84
Swin Transformer	93.71	90.20	91.00	93.14	0.81
**The proposed method**	**97.80**	**98.71**	**96.33**	**96.81**	**0.95**

**Table 4:** Performance evaluation of the proposed model using various optimizers.

Optimizer	ACC(%)	Pre(%)	Rec(%)	F1-score(%)	MCC
Adamax	80.13	74.37	79.84	83.20	0.63
RMSprop	86.70	89.00	82.91	85.43	0.70
Adadelta	87.34	86.16	89.00	88.41	0.74
Adam	89.81	82.00	92.10	88.64	0.75
Adagrad	91.32	90.50	92.62	91.54	0.87
**SGD**	**97.80**	**98.71**	**96.33**	**96.81**	**0.95**

**Table 5:** Ablation study of the proposed model components.

Model Variant	ACC(%)	Pre(%)	Rec(%)	F1-score(%)	MCC
Using ResNet50 only	79.62	72.03	69.56	74.70	0.68
Using DenseNet121 only	85.03	81.28	79.35	83.46	0.82
Using Swin Transformer only	93.75	90.22	91.06	93.18	0.81
Without Sobel filtering	94.00	91.55	93.20	92.11	0.82
Without DWT	94.93	92.11	94.40	93.14	0.85
Without AE	95.41	93.80	94.92	94.05	0.87
**The proposed method**	**97.80**	**98.71**	**96.33**	**96.81**	**0.95**

**Table 6:** Performance comparison between stacking and soft voting ensembles over 10 runs. Results presented as mean ± standard deviation. Statistical significance was assessed using a paired t-test.

Metric	Stacking (Mean ± Std)	Soft Voting (Mean ± Std)	P-Value
Acc	97.43 ± 0.13%	97.06 ± 0.10%	< 0.0010
Pre	98.12 ± 0.15%	97.70 ± 0.18%	< 0.0012
Rec	96.50 ± 0.17%	96.00 ± 0.16%	< 0.0010
F1-score	97.42 ± 0.14%	96.83 ± 0.15%	< 0.0010
AUROC	98.85 ± 0.14%	98.40 ± 0.15%	< 0.0011

**Table 7:** Performance metrics of the proposed model with 95% confidence intervals estimated via bootstrapping (N=1000). The confidence intervals indicate the statistical reliability of the reported metrics on the dataset.

Metric	Mean	Confidence Interval 95%
Acc	97.80%	97.20% - 98.30%
Pre	98.71%	98.20% - 99.10%
Rec	96.33%	95.70% - 96.90%
F1-score	96.81%	96.20% - 97.40%
AUROC	0.990	0.986 - 0.993
MCC	0.950	0.930 - 0.970

**Table 8:** Previous studies for PE classification. Here, AUC, CC, MIL, sen, spc, SSL, SPE-YOLO stand for area under the curve, conventional classification, multiple instance learning, sensitivity, specificity, self-supervised learning, and SE-Attention Prioritizes Features PE-You Only Look-Once respectively.

Study	Dataset	Architecture	Task Type	Findings
Condrea et al.^15^	RSNA	Dual-hup DNN with anatomical aware pretraining	PE detection from CT images	sen = 92.05%, spc = 96.17%
Ma et al.^26^	RSNA	Two phase multi task learning with interpretability(Grad-CAM, attention)	PE detection +localization +chronicity+ RV/LV ratio	AUROC = 0.93, sen = 86.02%
Khan et al.^27^	RSNA	DL framework based on DenseNet201 (feature extractor)+ customized fully connected layers	Multi-classification (PE classification into 9 classes)	Acc = 88.01%, sen = 88.00%, AUC = 0.90
Islam et al.^28^	CTPA dataset (specific dataset not mentioned)	Comparative study: CNNs vs ViTs; SSL vs supervised; transfer learning vs training from scratch; CC vs MIL	PE diagnosis(image-level and exam-level)	AUC = 0.96
Lynch et al.^32^	RSNA	PE-DeepNet: hybrid deep CNN with reduced parameters	PE classification	Acc = 94.21%
Suman et al.^36^	RSNA	Two stage attention-based CNN-LSTM network	PE detection+ type(chronic/acute)+ location(left/right/central)	AUC = 0.95
Wu et al.^35^	Tianjin internal(n=142) +RSNA test set(n=2000)	SPE-YOLO: YOLOv8 + P2 head+ SE-Attention+ ODconv for small PE detection	Small PE detection	sen = 90.71%, Acc = 86.45%
Mohammed et al.^20^	RSNA	EfficientNet-B7 + enhanced ViT with multi-task learning	Multi-task classification(PE detection, location, type)	AUC = 0.96
Cahan et al.^37^	Internal multimodal dataset(3D CTPA + clinical data)	Bilinear attention+ TabNet(structured + maging)	PE severity risk stratification (classification)	AUC = 0.96, sen = 90.00%, spc = 94.00%
**The proposed method**	RSNA	**Ensemble approach (ResNet50 + DenseNet121 + Swin Transformer)**	**Binary classification**	**Acc = 97.80%, AUROC = 0.99**

## Discussion

PE is a clot of blood that goes to the lungs and can cause considerable morbidity and mortality. Therefore, early diagnosis and therapy are critical. The most common procedure for diagnosing PE is CTPA images^59^. Human detection by radiologists increases the effort, requires more time, and needs experience for proper examination. An effective PE detection technique can help radiologists increase productivity by prioritizing urgent cases and speeding up at-risk patient diagnoses. For this target, we constructed a DL framework for PE detection. The proposed framework experimentally confirmed with a benchmark dataset of CT images divided into two classes: normal and PE. The suggested technique utilizes an ensemble of ResNet50, DenseNet121, and Swin Transformer for PE classification. The approach begins with a comprehensive preprocessing pipeline including AE-based dimensionality reduction, data augmentation, DWT, and Sobel filter for edge enhancement and noise reduction. Moreover, various optimizers were evaluated to optimize performance. This hybrid ensemble leverages the complementary strengths of CNNs for local feature extraction and Swin Transformer for capturing global context, leading to improved accuracy and robustness.

The main goal is to build an analysis framework that ensures high accuracy. To evaluate its performance, we conducted several experiments using metrics such as accuracy, precision, recall, F1-score, MCC, and ROC curves. To address the dataset imbalance, several data augmentation techniques (i.e., rotation,zoom, shear, and shifts. Additionally, vertical and horizontal flipping, along with gaussian blur were applied before feeding the dataset to the system. Additional comparison between the suggested model and the pre-trained DL-based models, including ResNet50, Vgg16, and DenseNet121, has been performed. We consolidated the parameters for all models by applying SGD optimizer, setting learning rate to $10^{-5}$, batch size of 32, and 20 epochs. As shown in Table 2, the proposed model emphasizes clear benefits over pre-trained models even with a low training time (20 epochs). Across all evaluation metrics, the proposed model showed better performance than all other models with peak values of 97.80%, 98.71%, 96.33%, 96.81%, and 0.95 for accuracy, precision, recall, F1-score, and MCC. In comparison, ResNet50, Vgg16, and DenseNet121 scored lower performance with accuracies of 79.64%, 82.39%, and 85.00%, respectively. While Vgg16 and DenseNet121 performed well in precision and recall, the proposed model provided better overall balance and performance.

Similarly, in the second experiment, the proposed model scored the highest performance across all evaluation metrics. The comparison was conducted between the suggested approach and the ViT models such as DeiT, PVT, and Swin Transformer. It recorded the highest scores of 97.80%, 98.71%, 96.33%, 96.81%, and 0.95 for accuracy, precision, recall, F1-score, and MCC. Table 3 summarizes the results of the second experiment. Although ViT models like DieT, PVT, and Swin Transformer showed competitive results with accuracies between 88.64% and 93.71%, they were outperformed by the proposed method in all aspects. To further investigate the proposed model’s performance, we evaluated it using different optimizers to evaluate their impact on the results. In the experiment with different optimizer, the proposed model attained the best performance using SGD optimizer an accuracy of 97.80%, and 98.71%, 96.33%, 96.81%, 0.95 for precision, recall, F1-score, and MCC. Other optimizers such as Adamax, resulted in the lowest performance with an accuracy of 80.13% and MCC of 0.63. RMSprop, Adadelta, and Adam produced moderate improvements, but none matched the performance of SGD. Adagrad scored better results than others with an accuracy of 91.32% and MCC of 0.87, but it still didn’t outperform SGD. Additionally, confusion matrices and ROC curves were calculated for this experiment as shown in Figs. 6, 7. In PE detection false negatives represent missed diagnosis which can lead to a fatal consequences. From Fig. 6, the proposed model demonstrated strong performance by correctly detecting the majority of PE cases (94.51%), thus minimizing the risk of missed diagnosis. Although false positives (0.57%) may result in additional diagnostic tests, their clinical impact is generally less severe. This trade-off allows the model to offer dependable support in clinical decision-making. Also, from Fig. 7, it scored an AUROC of 0.99. This value indicated outstanding performance, showing a highly accurate ability to distinguish between positive and negative classes.

To assess the effectiveness of the proposed stacking ensemble, we conducted a quantitative comparison with a baseline soft voting ensemble using the same base models. Each model was evaluated over 10 independent runs to account for performance variability. Table 6 summarizes the results, presenting the mean and standard deviation for each metric along with p-values derived from a paired t-test. The stacking method consistently outperformed soft voting across all key metrics including accuracy, precision,recall, and AUROC. Notably, the improvements were statistically significant with p-values less than 0.001 for all metrics indicating strong confidence that the observed gains are unlikely to be attributable to random variation. These findings confirm the superior performance and robustness of the proposed stacking strategy compared to standard fusion techniques. In addition, to further emphasize the statistical robustness of the proposed model’s performance, we calculated 95% confidence intervals (CIs) for the key evaluation metrics. These intervals were estimated using a bootstrap resampling method with 1000 iterations to provide reliable assessments of the variability across the dataset. As presented in Table 7 the model achieved an accuracy of 97.80% (95% CI: 97.20%−98.30%), precision of 98.71% (95% CI: 98.20%−99.10%), recall of 96.33% (95% CI: 95.70%−96.90%), F1-score of 96.81% (95% CI: 96.20%−97.40%), MCC of 0.950 (95% CI: 0.930-0.970), and an AUROC of 0.990 (95% CI: 0.986-0.993). These narrow confidence intervals indicate consistent and reliable performance, reinforcing the model’s capability to distinguish between the cases with and without PE. As highlighted in Table 8, the suggested approach outperformed previous studies in terms of performance. Generally, it is noted that the performance of the ensemble model which integrates ResNet50, DenseNet121, and Swin Transformer is better than that of other scenarios involving ablated models.Additionally, to enhance the model’s interpretability, Grad-CAM heatmaps were employed to visualize the regions that influenced the model’s decisions. Warmer colors (e.g., red) indicate areas of higher attention, while cooler colors (e.g., blue) denote less relevant regions. This visual explanation improves the transparency of the proposed model and may support clinical trust in its predictions. While Grad-CAM visualizations offer a degree of interpretability, their alignment with radiological reasoning has yet to be formally validated. Although many of the highlighted regions appear to correspond with PE-typical defects upon visual inspection, a structured clinician reader study is required to rigorously assess their reliability and clinical value.

The aforementioned results demonstrated that the proposed model can benefit healthcare systems by reducing consultation-related expenses, improving detection consistency, and minimizing the likelihood of disease-related fatalities. While the results demonstrate the effectiveness of the proposed ensemble approach for PE detection, it is important to emphasize the novel aspects that distinguish this work from existing studies. Unlike prior methods that often rely on simple decision-level fusion of CNNs and transformers, our approach introduces a stacked fusion layer that integrates feature representations at a deeper level, allowing for enhanced interaction between diverse model outputs. Moreover, the preprocessing pipeline applied in this study is unique in its combination of AE-based dimensionality reduction, DWT, and Sobel filtering. This hybrid pipeline improves feature quality by reducing noise and enhancing edge information, which is critical for accurate detection in medical tasks. Although the encouraging results, there are some limitations in our analysis design. One of the key limitations is that we trained and evaluated our model on just one benchmark dataset (RSNA). Therefore, incorporating external datasets from various centers would likely improve the reliability of our automated system. Additionally, our approach used only one type of input data: CT scans. In medical care, multiple sources of data are frequently used, such as clinical biomarkers. As a result, an update to the suggested technique might include various inputs and provide a more reliable prognosis at the patient stage. The proposed model showcased exceptional performance, achieving an accuracy of 97.80% and an AUROC of 0.99 on RSNA dataset, highlighting its strong potential for supporting clinical decision making in PE detection. These results suggest that the model could be a valuable tool for radiologists, especially in time-critical or high volume environments. While the outcomes are promising, we acknowledge the need for additional validation using more diverse and external datasets to ensure its reliability across various patient populations. This is a crucial step in the clinical validation process. We also anticipate future collaboration with clinicians to assess the model’s real-world integration and its usability in clinical workflows. Furthermore, while the current model is limited to binary PE detection (present/absent) future work could involve adapting the framework for multi-class classification based on embolus location(e.g.; central, segmental, subsegmental). This extension would enhance the clinical relevance of the model, as PE subclassification is critical for guiding appropriate treatment strategies. Achieving this would require access to more granularly labeled datasets as possibly integrating spatial localization mechanisms into the model architecture.

## Conclusion

In this study, we developed a hybrid ensemble model integrating Resnet50, DenseNet121, and Swin Transformer architectures for the early detection of PE from CT images. The proposed framework demonstrated superior diagnostic performance on the RSNA-STR dataset, highlighting its potential clinical values. However, these findings should be considered preliminary as external validation on diverse, independent datasets is essential to confirm generalizability and support clinical deployment. Future research will prioritize external validation across diverse populations and the incorporation of additional clinical data to improve the model’s generalizability and clinical utility. Additionally, evaluating the clinical trustworthiness of Grad-CAM visualizations through structured clinician reader studies will be a key focus in subsequent work.

## Data Availability

The dataset used is publicly available on https://www.kaggle.com/c/rsna-str-pulmonary-embolism-detection/data.

## References

[1] Li, H., Hu, Z. & Hu, M. Research on attention mechanism based assisted diagnosis of pulmonary embolism. In *Chinese Intelligent Systems Conference*, 27–37 (Springer, 2023).

[2] Su, H. et al. Detection of pulmonary embolism severity using clinical characteristics, hematological indices, and machine learning techniques. *Front. Neuroinformatics* **16**, 1029690 (2022). 10.3389/fninf.2022.1029690 PMC9800512 36590906

[3] Dua, R., Ronald Wallace, G., Chotso, T. & Francis Densil Raj, V. Classifying pulmonary embolism cases in chest ct scans using vgg16 and xgboost. In *Intelligent Communication Technologies and Virtual Mobile Networks: Proceedings of ICICV 2022*, 273–292 (Springer, 2022).

[4] Goldhaber, S. Z. & Bounameaux, H. Pulmonary embolism and deep vein thrombosis. *The Lancet* **379**, 1835–1846 (2012). 10.1016/S0140-6736(11)61904-1 22494827

[5] Rivas, L. F. Clinical characterization of patients with venous thromboembolic disease in 2 reference centers in el salvador. *Blood* **142**, 5555 (2023).

[6] Sukumar, S., Harish, A., Shahina, A., Sanjana, B. & Khan, A. N. Deep learning based pulmonary embolism detection using convolutional feature maps of ct pulmonary angiography images. *Procedia Comput. Sci.* **233**, 317–326 (2024).

[7] Yang, X. et al. A two-stage convolutional neural network for pulmonary embolism detection from ctpa images. *IEEE Access* **7**, 84849–84857 (2019).

[8] Agnelli, G. & Becattini, C. Anticoagulant treatment for acute pulmonary embolism: a pathophysiology-based clinical approach. *Eur. Respir. J.* **45**, 1142–1149 (2015). 25700388 10.1183/09031936.00164714

[9] Barco, S. et al. Age-sex specific pulmonary embolism-related mortality in the usa and canada, 2000–18: an analysis of the who mortality database and of the cdc multiple cause of death database. *The Lancet Respir. Medicine* **9**, 33–42 (2021). 10.1016/S2213-2600(20)30417-3 PMC7550106 33058771

[10] Cohen, A. et al. The number of vte events and associated morbidity and mortality. *Thromb Haemost* **98**, 756–764 (2007). 17938798 10.1160/TH07-03-0212

[11] Lee, L. C. & Shah, K. Clinical manifestation of pulmonary embolism. *Emerg. Medicine Clin.* **19**, 925–942 (2001). 10.1016/s0733-8627(05)70227-3 11762280

[12] Hendriks, B. M. et al. Optimizing pulmonary embolism computed tomography in the age of individualized medicine: a prospective clinical study. *Investig. Radiol.* **53**, 306–312 (2018). 29438139 10.1097/RLI.0000000000000443

[13] Donohoo, J. H., Mayo-Smith, W. W., Pezzullo, J. A. & Egglin, T. K. Utilization patterns and diagnostic yield of 3421 consecutive multidetector row computed tomography pulmonary angiograms in a busy emergency department. *J. computer assisted tomography* **32**, 421–425 (2008). 10.1097/RCT.0b013e31812e6af3 18520550

[14] Hoeper, M. M. et al. Chronic thromboembolic pulmonary hypertension. *The Lancet Respir. Medicine* **2**, 573–582 (2014). 10.1016/S2213-2600(14)70089-X 24898750

[15] Condrea, F. et al. Anatomically aware dual-hop learning for pulmonary embolism detection in ct pulmonary angiograms. *Comput. Biol. Medicine* **174**, (2024). 10.1016/j.compbiomed.2024.108464 38613894

[16] Foti, G. et al. Identification of pulmonary embolism: diagnostic accuracy of venous-phase dual-energy ct in comparison to pulmonary arteries ct angiography. *Eur. Radiol.* **31**, 1923–1931 (2021). 32965572 10.1007/s00330-020-07286-7

[17] Shen, J. et al. Massive external validation of a machine learning algorithm to predict pulmonary embolism in hospitalized patients. *Thromb. Res.* **216**, 14–21 (2022). 35679633 10.1016/j.thromres.2022.05.016

[18] Özkan, H., Osman, O., Şahin, S. & Boz, A. F. A novel method for pulmonary embolism detection in cta images. *Comput. methods and programs in biomedicine* **113**, 757–766 (2014). 10.1016/j.cmpb.2013.12.014 24440133

[19] Tajbakhsh, N., Shin, J. Y., Gotway, M. B. & Liang, J. Computer-aided detection and visualization of pulmonary embolism using a novel, compact, and discriminative image representation. *Med. image analysis* **58**, (2019). 10.1016/j.media.2019.101541 PMC6815717 31416007

[20] Mohammed, A. N., Kuang, H. & Wang, J. Vit-based multi-task learning method for pulmonary embolism detection, localization, and type classification. In *International Conference on Intelligent Computing*, 467–478 (Springer, 2024).

[21] Ahmed, M. M. et al. Brain tumor detection and classification in mri using hybrid vit and gru model with explainable ai in southern bangladesh. *Sci. Reports* **14**, 22797 (2024). 10.1038/s41598-024-71893-3 PMC11445444 39354009

[22] Hossain, M. M. et al. A novel hybrid vit-lstm model with explainable ai for brain stroke detection and classification in ct images: A case study of rajshahi region. *Comput. Biol. Medicine* **186**, (2025). 10.1016/j.compbiomed.2025.109711 39847947

[23] Deng, S. et al. Deep learning in digital pathology image analysis: a survey. *Front. medicine* **14**, 470–487 (2020). 10.1007/s11684-020-0782-9 32728875

[24] Foley, R. W. et al. Automated calculation of the right ventricle to left ventricle ratio on ct for the risk stratification of patients with acute pulmonary embolism. *Eur. Radiol.* **31**, 6013–6020 (2021). 33459854 10.1007/s00330-020-07605-y

[25] Tajbakhsh, N., Gotway, M. B. & Liang, J. Computer-aided pulmonary embolism detection using a novel vessel-aligned multi-planar image representation and convolutional neural networks. In *Medical Image Computing and Computer-Assisted Intervention–MICCAI 2015: 18th International Conference, Munich, Germany, October 5-9, 2015, Proceedings, Part II 18*, 62–69 (Springer, 2015).

[26] Ma, X., Ferguson, E. C., Jiang, X., Savitz, S. I. & Shams, S. A multitask deep learning approach for pulmonary embolism detection and identification. *Sci. Reports* **12**, 13087 (2022). 10.1038/s41598-022-16976-9 PMC9338063 35906477

[27] Khan, M. et al. Iomt-enabled computer-aided diagnosis of pulmonary embolism from computed tomography scans using deep learning. *Sensors* **23**, 1471 (2023). 36772510 10.3390/s23031471 PMC9921395

[28] Islam, N. U., Gehlot, S., Zhou, Z., Gotway, M. B. & Liang, J. Seeking an optimal approach for computer-aided pulmonary embolism detection. In *Machine Learning in Medical Imaging: 12th International Workshop, MLMI 2021, Held in Conjunction with MICCAI 2021, Strasbourg, France, September 27, 2021, Proceedings 12*, 692–702 (Springer, 2021). 10.1007/978-3-030-87589-3_71 PMC9184235 35695860

[29] Yuan, H., Shao, Y., Liu, Z. & Wang, H. An improved faster r-cnn for pulmonary embolism detection from ctpa images. *IEEE Access* **9**, 105382–105392 (2021).

[30] Huhtanen, H. et al. Automated detection of pulmonary embolism from ct-angiograms using deep learning. *BMC Med. Imaging* **22**, 43 (2022). 35282821 10.1186/s12880-022-00763-z PMC8919639

[31] Huang, S.-C. et al. Penet-a scalable deep-learning model for automated diagnosis of pulmonary embolism using volumetric ct imaging. *NPJ digital medicine* **3**, 61 (2020). 32352039 10.1038/s41746-020-0266-y PMC7181770

[32] Lynch, D. & Suriya, M. Pe-deepnet: A deep neural network model for pulmonary embolism detection. *Int. J. Intell. Networks* **3**, 176–180 (2022).

[33] Djahnine, A. et al. Detection and severity quantification of pulmonary embolism with 3d ct data using an automated deep learning-based artificial solution. *Diagn. Interv. Imaging* **105**, 97–103 (2024). 38261553 10.1016/j.diii.2023.09.006

[34] Guo, J. *et al.* Aanet: artery-aware network for pulmonary embolism detection in ctpa images. In *International Conference on Medical Image Computing and Computer-Assisted Intervention*, 473–483 (Springer, 2022).

[35] Wu, H. et al. Spe-yolo: A deep learning model focusing on small pulmonary embolism detection. *Comput. Biol. Medicine* **184**, (2025). 10.1016/j.compbiomed.2024.109402 39536384

[36] Suman, S. *et al.* Attention based cnn-lstm network for pulmonary embolism prediction on chest computed tomography pulmonary angiograms. In *Medical Image Computing and Computer Assisted Intervention–MICCAI 2021: 24th International Conference, Strasbourg, France, September 27–October 1, 2021, Proceedings, Part VII 24*, 356–366 (Springer, 2021).

[37] Cahan, N. et al. Multimodal fusion models for pulmonary embolism mortality prediction. *Sci. Reports* **13**, 7544 (2023). 10.1038/s41598-023-34303-8 PMC10170065 37160926

[38] RSNA, S. Pulmonary embolism detection. *Kaggle*. https://www.kaggle.com/c/rsna-str-pulmonary-embolism-detection/. Accessed September **30** (2020).

[39] Colak, E. *et al.* The rsna pulmonary embolism ct dataset. *Radiol. Artif. Intell. ***3**, e200254 (2021). 10.1148/ryai.2021200254 PMC8043364 33937862

[40] Masutani, Y., MacMahon, H. & Doi, K. Computerized detection of pulmonary embolism in spiral ct angiography based on volumetric image analysis. *IEEE Transactions on Med. Imaging* **21**, 1517–1523 (2002). 10.1109/TMI.2002.806586 12588035

[41] Chan, H., Hadjiiski, L., Zhou, C. & Sahiner, B. Pulmonary embolism and deep vein thrombosis. *Acad. Radiol* **15**, 535–555 (2008). 18423310 10.1016/j.acra.2008.01.014 PMC2800985

[42] Chilukuri, P., Kumar, J. A., Anusuya, R. & Prabhu, M. R. Auto encoders and decoders techniques of convolutional neural network approach for image denoising in deep learning. *J. Pharm. Negat. Results* **13**, 1036–1040 (2022).

[43] Siddalingappa, R. & Kanagaraj, S. Anomaly detection on medical images using autoencoder and convolutional neural network. *Int. J. Adv. Comput. Sci. Appl.* (2021).

[44] Mustafa, W. A. *et al.* Image enhancement based on discrete cosine transforms (dct) and discrete wavelet transform (dwt): a review. In *IOP Conference Series: Materials Science and Engineering*, vol. 557, 012027 (IOP Publishing, 2019).

[45] Gungor, M. A. A comparative study on wavelet denoising for high noisy ct images of covid-19 disease. *Optik* **235**, 166652 (2021). 33688101 10.1016/j.ijleo.2021.166652 PMC7931685

[46] Alickovic, E., Kevric, J. & Subasi, A. Performance evaluation of empirical mode decomposition, discrete wavelet transform, and wavelet packed decomposition for automated epileptic seizure detection and prediction. *Biomed. signal processing control* **39**, 94–102 (2018).

[47] Chang, S. G., Yu, B. & Vetterli, M. Adaptive wavelet thresholding for image denoising and compression. *IEEE transactions on image processing* **9**, 1532–1546 (2000). 18262991 10.1109/83.862633

[48] Trivedi, G. & Sanghavi, R. Fusesharp: A multi-image focus fusion method using discrete wavelet transform and unsharp masking. *J. applied mathematics & informatics* **41**, 1115–1128 (2023).

[49] Acharjya, P. P., Das, R. & Ghoshal, D. A study on image edge detection using the gradients. *Int. J. Sci. Res. Publ.* **2**, 1–5 (2012).

[50] Li, Z., Jin, H. & Xing, X. Edge detection algorithm of fractional order sobel operator for integer order differential filtering. *Comput. Eng. Appl* **54**, 179–184 (2018).

[51] Rana, R. & Verma, A. Comparison and enhancement of digital image by using canny filter and sobel filter. *IOSR J. Comput. Eng.* **16**, 06–10 (2014).

[52] Zhang, K., Zhang, Y., Wang, P., Tian, Y. & Yang, J. An improved sobel edge algorithm and fpga implementation. *Procedia Comput. Sci.* **131**, 243–248 (2018).

[53] Aina, J., Akinniyi, O., Rahman, M. M., Odero-Marah, V. & Khalifa, F. A hybrid learning-architecture for mental disorder detection using emotion recognition. *IEEE Access* (2024). 10.1109/access.2024.3421376 PMC11270886 39054996

[54] Parvathavarthini, M. K. & Anandan, R. Optimizing medical diagnostics: Improving ct imaging with swin transformer and attention networks. *Educ. Adm. Theory Pract.* **30**, 9203–9208 (2024).

[55] Liu, Z. *et al.* Swin transformer: Hierarchical vision transformer using shifted windows. In *Proceedings of the IEEE/CVF international conference on computer vision*, 10012–10022 (2021).

[56] Wolpert, D. H. *Stacked generalization. Neural networks* **5**, 241–259 (1992).

[57] Paymode, A. S. & Malode, V. B. Transfer learning for multi-crop leaf disease image classification using convolutional neural network vgg. *Artif. Intell. Agric.* **6**, 23–33 (2022).

[58] Zhang, R. *et al.* Enhancing medical image classification with context modulated attention and multi-scale feature fusion. *IEEE Access* (2025).

[59] Haque, M. A., Shome, A. & Bairagi, A. K. Diagnosis of pulmonary embolism from ct pulmonary angiogram by using deep transfer learning and sobel filter. In *2024 15th International Conference on Computing Communication and Networking Technologies (ICCCNT)*, 1–7 (IEEE, 2024).

